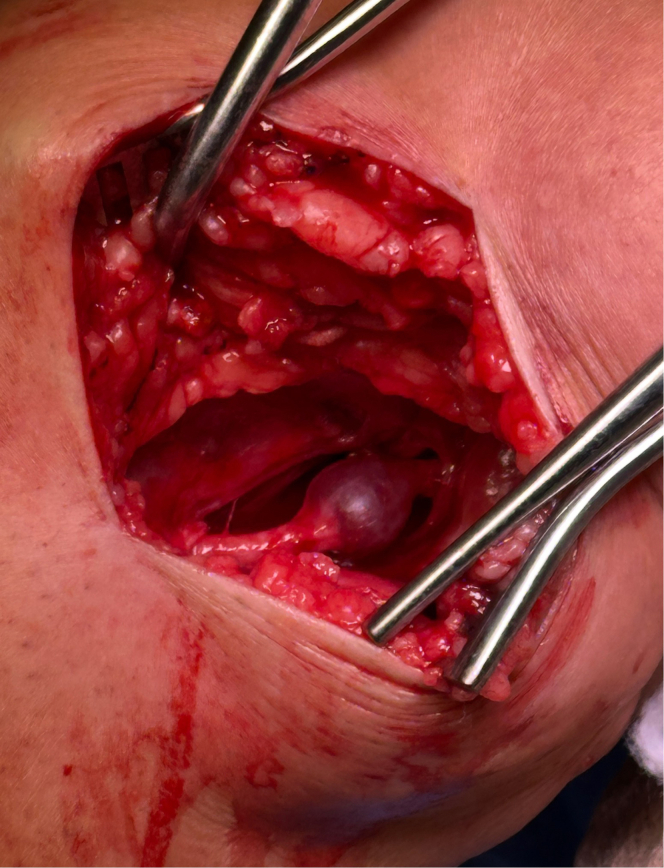# Repair of a popliteal vein aneurysm following a torn meniscus

**DOI:** 10.1016/j.jvsv.2025.102301

**Published:** 2025-08-11

**Authors:** Jack Petroski, Raj Patel, Gurpreet Singh, Raghuram Gorti

**Affiliations:** a Touro College of Osteopathic Medicine, Middletown, NY; b Garnet Health Medical Center, Department of General Surgery, Middletown, NY

A 58-year-old Caucasian woman, was gravida three, para two-zero-two-one, with a history of asthma, degenerative joint disease, complex regional pain syndrome, gastritis, a right knee meniscal tear 3 months prior, and a 40 pack-year smoking history, was referred to vascular surgery for evaluation of a posterior knee mass incidentally found on magnetic resonance imaging. She had presented with several months of progressive right knee swelling and discomfort. She denied claudication, rest pain, or tissue loss. Physical examination revealed diffuse swelling and pinpoint inferomedial tenderness without overlying erythema. Magnetic resonance imaging showed a 1.4 × 0.9 × 1.6 cm posterior knee mass, and duplex ultrasound confirmed a popliteal vein aneurysm without thrombus or arterial involvement (*A*/Cover). She underwent tangential aneurysmectomy with lateral venorrhaphy (*B*) and was discharged on apixaban. Follow-up was unremarkable, with only a physical examination performed; no additional imaging was obtained. Meniscal repair is still anticipated.

Popliteal vein aneurysms are rare, with only 173 confirmed cases reported in the literature.^1^ Although often asymptomatic, they carry the risk of deep vein thrombosis, rupture, or pulmonary embolism. Proposed etiologies include congenital venous wall weakness, trauma, or chronic venous hypertension. Diagnosis relies on duplex ultrasound to assess morphology, size, and thrombus burden. Surgical repair is favored in symptomatic cases or when aneurysms are large (>20 mm) or saccular due to the heightened risk of embolization. In this case, operative intervention was performed due to persistent symptoms and concern for thromboembolic complications. Popliteal vein aneurysms, although uncommon, should be considered in the differential diagnosis of popliteal fossa masses to ensure timely recognition and prevent potentially serious outcomes.

**Figure:**
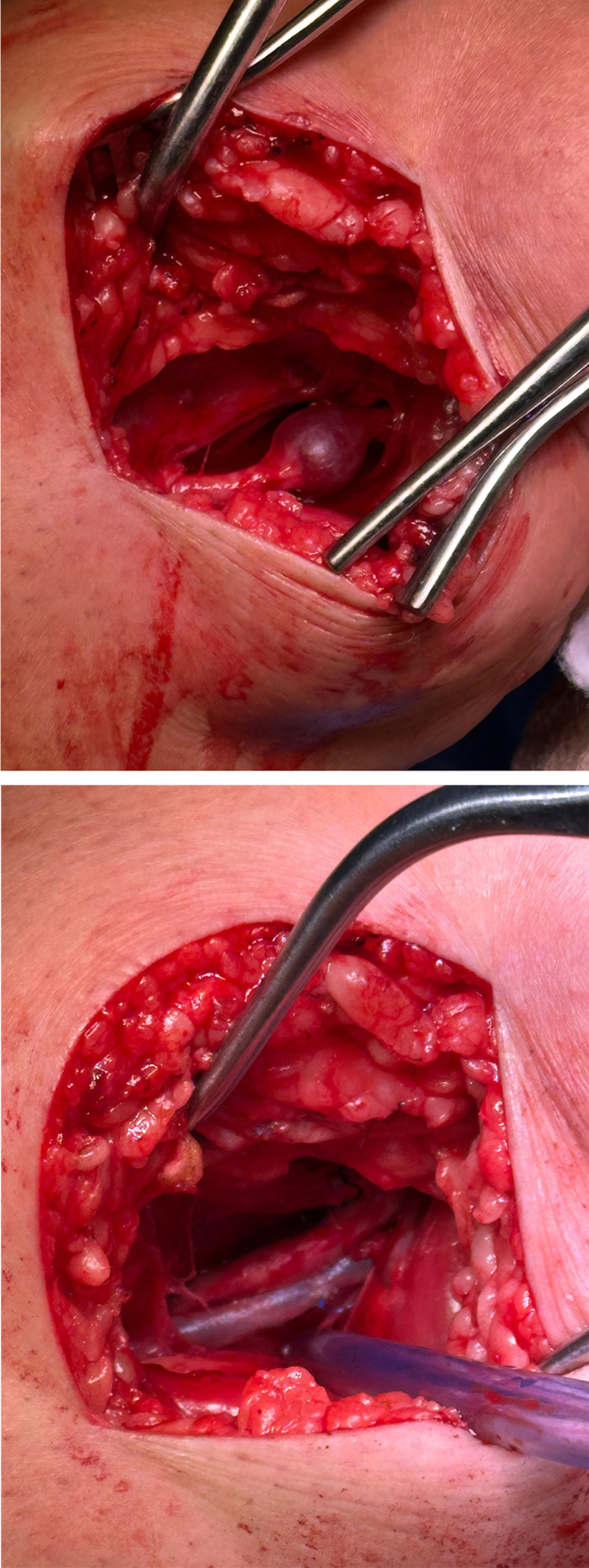

## Funding

None.

## Disclosures

None.

## Figures and Tables

**Cover image:**